# Supplementary material for: COVID-19 and Bangladeshi health professionals: Infection status, vaccination and its immediate health consequences
Source: PLoS One. 2022 Nov 15;17(11):e0277022. doi: 10.1371/journal.pone.0277022 (PMC9665383; doi:10.1371/journal.pone.0277022)
Supplement: Attachment — Submitted filename: Considerations.docx [file pone.0277022.s002.docx]

“COVID-19 and Bangladeshi Health Professionals: Infection status, vaccination and its immediate health consequences”

Considerations:Thank you for the opportunity to review this article.

Overall:The article is well written and substantiated. I congratulate the authors for their initiative.

Title: Well presented

Abstract:

The abstract is longer than 300 words

Introduction:

On line 89 the acronym SAGE was placed without describing what it means. The first time an acronym is recorded it should say what it means.

I would add in line 64, the number of active health workers in Bangladesh, because by putting only the number of deaths you don't have the dimension of how many died, if it was a lot or a little? This enriches the argument about how serious the number of deaths among health workers is.

In line 78 : "through then, we could eradicate small pox & nearly eliminate the wild-polio.[14]" Place the quote before the period.

Method:

A detailed description is needed, especially of the reliability and validity of the questionnaire.

Further detail the eligibility and exclusion criteria for participants.

How was the question of COVID-19 infection, was it considered what types of testing? How did you guys consider this data?

Results:

Better specify what Immediate health consequences of COVID-19 vaccination would be.

Discussion:

Well presented

Conclusion:

Resume in the conclusion what the immediate health consequences of having had an infection and being vaccinated are for the health of the health care worker. What happens if the professional is infected? And what are the repercussions of vaccination in this professional category?

This is the moment to value your findings.

Translated with www.DeepL.com/Translator (free version)
