# Supplementary material for: COVID-19 and Bangladeshi health professionals: Infection status, vaccination and its immediate health consequences
Source: PLoS One. 2022 Nov 15;17(11):e0277022. doi: 10.1371/journal.pone.0277022 (PMC9665383; doi:10.1371/journal.pone.0277022)
Supplement: Attachment — Submitted filename: Response to reviewer.docx [file pone.0277022.s003.docx]

| **Reviewer's Comment** | **Response** |
| --- | --- |
| **Reviewer 1** | |
| **Abstract** | |
| 1. Introduction: The justification should be more succinct as it is in the body of the literature. The objective should be explicitly described. Consider my suggested objective in the manuscript. | All indications are addressed in line numbers 22 to 28  We did not find any suggested objective. However, we modified it also in 26-28 lines. |
| 2. Method: You need to remove the sentence on the composition of the health workers. Rather, you should have the information on the content of the questionnaire in this section. | Removed and included information from questionnaire in line 32 and 33. |
| 3. Results: You need to add the 95% CI for the infection rates. | Added in line 38, 40, 43 & 44 |
| **Body of the manuscript** |  |
| 4. Line 85, please write out the full meaning of SAGE if it appearing here for the first time. | Added in line 99. |
| 5. Line 103, what do you mean by ‘novel professional’? Please this should be clearer or choose the appropriate word. | Modified in line 68-71 |
| 6. Please add the objective of this study to the end of the introduction section.  The justification/rationale for this study within the introduction is too lengthy for an article. Please endeavor to summarize it. | Objective already mentioned in line 118-121;  Justification is modified and concised. |
| 7. You had no information on the study setting; the readers may be interested in this. The prevention measures prevalent in the hospital, presence of a functional IPC team, nature of COVID-19 immunization activities, actions for AEFIs following Covid-19 vaccination etc. You can cite reference, if it has been previously published.  Mention the inclusion and exclusion criteria for the respondents. | Included in the manuscript in line 132, 135-138;  Inclusion and exclusion criteria are mentioned in line 160-162; |
| Mention the sampling procedure for selecting the hospitals. | Mentioned in line 135-38 and 158-60 |
| Line 135, p=0.41 from the reference cited, and not p=0.59, please replace. | Modified in line 150 |
| How did you check for duplicates of entries since it was an online based questionnaire?  You need to report the source of the 10 respondents used for the pretest. | Mentioned in line 165-67;  Information regarding pretest mentioned in line 184-86; |
| It is not clear how the infection status of the respondents was determined? Please explain or cite reference, if it has been previously published. | Included in line 189. |
| Results: Add your response rate (316/372) 84.9%. | Added in line 206-07. |
| Line 183; add the mean age of the respondents or as the case may be. Unless, it has been earlier published. | Added in 208. |
| Line 229-234, report only the adjusted Odds Ratio and its 95% CIs. | Incorporated |
| Discussion: Line 255-264, the first paragraph of the discussion should start with stating what the aim of the work was and a summary of your findings. | Incorporated in line 283-289 |
| Your recommendation still sounds like the rationale for the study. Please rephrase appropriately based on your conclusion. | Modified in the conclusion |
| You had no statement on limitation, especially being an online-based questionnaire, likewise on the generalizability of your findings. Please can you explain this? Otherwise, address them in the body of the manuscript. | Added in line 351-53 |
| A lot of grammatical errors please address them. I was able to address some in the manuscript. | Already addressed |
| **Reviewer 2** | |
| Abstract: The abstract is longer than 300 words | Incorporated |
| On line 89 the acronym SAGE was placed without describing what it means. The first time an acronym is recorded it should say what it means. | Added in line 99. |
| I would add in line 64, the number of active health workers in Bangladesh, because by putting only the number of deaths you don't have the dimension of how many died, if it was a lot or a little? This enriches the argument about how serious the number of deaths among health workers is. | Incorporated in line 68-71. |
| In line 78: "through then, we could eradicate small pox & nearly eliminate the wild-polio.[14]" Place the quote before the period. | Modified in line 86-88. |
| A detailed description is needed, especially of the reliability and validity of the questionnaire. | Mentioned in line 84. |
| Further detail the eligibility and exclusion criteria for participants. | Added in line 160-62 |
| How was the question of COVID-19 infection, was it considered what types of testing? | Yes, mentioned in line 189 |
| How did you guys consider this data? | Quantitative |
| Results: Better specify what Immediate health consequences of COVID-19 vaccination would be. | Modified and included in line 227-230. |
| Resume in the conclusion what the immediate health consequences of having had an infection and being vaccinated are for the health of the health care worker. What happens if the professional is infected? And what are the repercussions of vaccination in this professional category? | Incorporated in the conclusion |
| **Editor's Comment** | |
| Minor grammatical errors throughout the manuscript require correction. | Already addressed |
